# Comparison of Functional Movement Screen, Star Excursion Balance Test, and Physical Fitness in Junior Athletes with Different Sports Injury Risk

**DOI:** 10.1155/2020/8690540

**Published:** 2020-03-25

**Authors:** Wen-Dien Chang, Li-Wei Chou, Nai-Jen Chang, Shuya Chen

**Affiliations:** ^1^Department of Sport Performance, National Taiwan University of Sport, Taichung, Taiwan; ^2^Department of Physical Medicine and Rehabilitation, China Medical University Hospital, Taichung, Taiwan; ^3^Department of Physical Therapy and Graduate Institute of Rehabilitation Science, China Medical University, Taichung, Taiwan; ^4^Department of Rehabilitation, Asia University Hospital, Taichung, Taiwan; ^5^Department of Sports Medicine, Kaohsiung Medical University, Kaohsiung, Taiwan

## Abstract

**Objectives:**

The aim of this study was to assess the relationships between functional movement screen (FMS), star excursion balance test (SEBT), agility *T* test, and vertical jump test scores and sports injury risk in junior athletes. We compared these assessments and the differences between groups with high and low risks of sports injury. *Subjects and Methods*. Eleven volleyball, 12 basketball, and 9 handball athletes were recruited. All participants followed the routine training in school sports teams. Weekly training schedules followed a similar pattern. The 32 junior athletes (age = 16.06 ± 0.21 years; height = 167.28 ± 6.32 cm; and body mass = 68.45 ± 9.67 kg) were assessed using the FMS, SEBT, agility *T* test, and vertical jump test in random order. The correlations of composite and individual item scores of these assessments were analyzed, and the differences between groups with high and low risks of sports injury were compared.

**Results:**

All participants completed the study protocol. No significant differences were observed between FMS, SEBT, agility *T* test, and vertical jump test scores in groups with high and low risks of sports injuries. Fair and moderate-to-good correlations were observed for anterior reach maximum of SEBT and deep squat (*r* = 0.47, *P* = 0.02) as well as inline lunge (*r* = 0.53, *P* = 0.01) of FMS. The hurdle step of FMS also exhibited moderate-to-good (*r* = 0.52, *P* = 0.01) and fair (*r* = 0.42, *P* = 0.04) correlations with posterior medial and posterior lateral reach maximum of SEBT, respectively. A fair correlation was observed between posterior lateral reach maximum of SEBT and rotary stability of FMS (*r* = −0.23, *P* = 0.03). Fair and moderate-to-good correlations were identified for agility *T* test and maximum anterior reach in the SEBT (*r* = −0.42, *P* = 0.04) and trunk stability push-up in the FMS, respectively (*r* = −0.57 and *P* = 0.006).

**Conclusions:**

Junior athletes with a high risk of sports injury did not exhibit differences in terms of FMS, SEBT, and physical fitness test scores. Deep squat, hurdle step, inline lunge, and rotary stability scores in the FMS were correlated with the item scores in the SEBT, which may be due to the use of similar movement patterns. Scores for anterior reach maximum in the SEBT and trunk stability push-up in the FMS were correlated with agility *T* test scores, suggesting a similar task requirement of trunk stability and dynamic weight shifting ability.

## 1. Introduction

Owing to the growing numbers of sports injuries, the American Academy of Pediatrics has highlighted the potential risks of sports specialization and high-intensity training in young athletes [1]. Young athletes chosen in talent programs often have the opportunity to participate in various exercises and multiple competition levels [2]. Such early motor specialization was associated with an increased risk of acute and substantial injuries and an increased risk of injuries from overuse in the top 10% of athletes in a self-assessment group [3]. Because sports specialization and intensive training in junior athletes may affect musculoskeletal conditions, predicting sports injuries in this age group is imperative.

Functional movement and sports performance tests are used to assess an athlete's conditions and prevent sport-related injuries [4]. These tests could also be used as clinical tests to predict the risk of sports injury, because poor physical fitness, improper movement pattern, and insufficient sensorimotor control are vital factors in sports injuries [4, 5]. Clinical screening tests, such as the functional movement screen (FMS), star excursion balance test (SEBT), and agility and muscle power tests, are commonly used to assess sports performance and injury prevention [6]. The FMS is designed to identify motion deficits and body asymmetry and can assess general musculoskeletal conditions to predict injury risk [7]. This test can predict injuries with high specificity and exhibits moderate interrater reliability [8]. The SEBT assessed dynamic balance and physical performance [7]. It identifies the risk of sport-related injuries of the lower extremities and exhibits high interrater reliability [9]. The agility and muscle power tests are used to evaluate sports performance. Caswell et al. investigated the association between sports injury and physical performance in American youth football teams [10]. They concluded that a complex relationship exists between the agility and muscle power of an athlete in terms of their movements and rates of injury occurrence [10]. These four clinical screening tests could help identify the risk of injury and contribute to the formulation of a successful sports injury prevention strategy.

To the best of our knowledge, FMS and SEBT are functional assessment tools with some specific relevance [11]. However, information regarding a potential relationship between these assessments and physical fitness tests (i.e., agility and muscle power) is still insufficient. Additionally, the FMS and SEBT are used to predict the risk of sports injury occurrence. Currently, no research is available on the differences in FMS, SEBT, and physical fitness test results (i.e., agility and muscle power) between groups with high and low risks of sports injury. We hypothesized that correlations exist among these functional assessment tools and different sports injury risks. Therefore, the aim of this study was to investigate the relationships between FMS, SEBT, and physical fitness test results (i.e., agility and muscle power tests) and examine differences in these assessments in groups with high and low risks of sports injury.

## 2. Methods

### 2.1. Study Design

This observational study was conducted in accordance with current national and international laws and regulations governing the use of human subjects (Declaration of Helsinki II). It was approved by the Institutional Review Board of AT Hospital. Participants were informed of study procedures prior to their participation.

### 2.2. Participants

In this study, participants were junior athletes recruited from school sports teams. Inclusion criteria were as follows: being currently engaged in full sport participation, having a sport training history of more than 3 years, and being able to complete the study process. Exclusion criteria included suffering from acute sports injures and losing time for sport participation. The demographic and anthropometric characteristics of participants were recorded. All participants followed the routine training in school sports teams. Weekly training schedules were similar in the terms. The sessions contained fitness training for two half days per week and sport skill training for three half days per week. Both trainings were alternated in a week. The study procedure started after the academic year, and the assessments were performed before the start of sports competition season. Following the precedence of a study by Smith et al. [12], the sample size was estimated at a minimum of 19 participants. Eleven volleyball, 12 basketball, and 9 handball junior athletes were recruited. A total of 32 junior athletes (age = 16.06 ± 0.21 years; height = 167.28 ± 6.32 cm; body mass = 68.45 ± 9.67 kg) completed the study procedure.

### 2.3. Procedures

The four assessments (i.e., the FMS, SEBT, and agility and muscle power tests) were conducted in a randomized order. A dynamic stretching exercise as a warm-up was performed prior to the assessments. Both a 30-second break between testing movements and a 10-minute break between each assessment were employed. The participants performed each assessment three times, and all assessments were conducted by a physical therapist.

### 2.4. Assessments

#### 2.4.1. Functional Movement Screen

The FMS™ (https://www.FunctionalMovement.com, Danville, VA, USA) involves seven components: deep squat, hurdle step, inline lunge, shoulder mobility, active straight-leg raise, trunk stability push-up, and rotary stability. The score for each item ranges from 0 to 3, with 0 indicating pain, 1 indicating a noncompleted skill performance, 2 indicating a skill performance with compensation, and 3 indicating a skill performance without compensation. The maximum score for the FMS is 21, and a total score lower than or equal to 14 points on the FMS indicates a greater possibility of sustaining a sports injury [13]. The FMS has exhibited excellent interrater reliability (0.97) for examining adolescents [14].

#### 2.4.2. Star Excursion Balance Test

The SEBT is a balance test in which participants are asked to perform a single-leg standing test. In this study, the Y Balance Test™ (https://www.FunctionalMovement.com, Danville, VA, USA) was used to assess balance in three directions. Participants reached in the anterior, posteromedial, and posterolateral directions as far as possible, and the maximum distance was recorded in each direction. Three trials were performed, and each participant's limb length, measured from the anterior superior iliac spine to the medial malleolus, was recorded. The maximum distance of each direction was then divided by the participant's limb length. A difference in the anterior distance between both lower extremities greater than 4 cm indicates a high risk of sustaining a lower extremity injury [15]. In high school athletes, the SEBT exhibits good-to-excellent test-retest reliability (interrater reliability = 0.89‐0.93) for these three directions [16].

#### 2.4.3. Agility *T* Test

The agility *T* test is a running test in which four cones are arranged in a T shape. In this study, three cones were placed 4.57 m apart in a straight line. The starting cone was placed 9.14 m away, perpendicularly extending to the middle cone. Participants were asked to accelerate to touch each cone base and run forwards, laterally, and backwards between the cones as fast as possible. An electronic timing system (T-Test Agility Timing Systems, You-Shang Technical Corp., Taiwan) was used to record times in the test. This test has good test-retest reliability (intraclass correlation coefficient = 0.94) for assessing agility in running performance [17].

#### 2.4.4. Vertical Jump

A vertical jump test was used to assess the muscle power of participants' lower extremities. Participants were asked to touch a provided vane. They started in a standing position and then flexed their knees and hips to jump immediately. Vertical jumping height was calculated using Vertec vertical jump meter (Sports Imports Incorporated, Columbus, OH, USA). The test-retest trial has good reliability (ICC = 0.97) [18].

#### 2.4.5. Injury Risk Determination

FMS and SEBT scores have been used to predict sports injury risk in some studies [13, 15]. The sports injury risk cutoff points were 14 for total FMS score and a difference in the anterior distance of 4 cm in the SEBT, respectively. Total score of FMS > 14 or anterior distance difference distance of SEBT < 4 cm of SEBT was interpreted as low risk of sports injury. Oppositely, total FMS scores ≤ 14 or anterior distance difference of SEBT ≥ 4 cm was interpreted as indicating the group with a high risk of sports injury. Values from FMS, SEBT, agility *T* test, and vertical jump tests were compared in groups with high and low risks of sports injury.

### 2.5. Statistical Analysis

All data were analyzed using SPSS software (version 21; SPSS Inc., Chicago, IL., USA). Participant demographics (age, height, body mass, and sport) were calculated and reported as the mean ± standard deviation. Spearman correlation was used to assess the relationship among the FMS, SEBT, agility *T* test, and vertical jump test. The relationship between individual items of each assessment was analyzed. Correlation coefficients were established as low (*r* < 0.25), fair (0.25 ≤ *r* < 0.50), moderate-to-good (0.50 ≤ *r* < 0.75), and good-to-excellent (*r* ≥ 0.75). The Shapiro–Wilk test was used to perform normality analysis, and the result indicated a nonnormal distribution. Therefore, a nonparametric approach was used for analyzing the data. The Mann–Whitney *U* test was used to compare differences in the FMS, SEBT, agility *T* test, and vertical jump test between groups with high and low risks of sports injury. A two-tailed test was used, and the *α* level was 0.05.

## 3. Results

The scores of FMS, SEBT, agility *T* test, and vertical jump for all participants are shown in Table 1. As shown in Table 2, no significant differences were observed in the FMS, SEBT, agility *T* test, and vertical jump between high risk of injury group (total score of FMS ≤ 14, *n* = 20; anterior distance difference of SEBT ≥ 4 cm, *n* = 18) and low risk group (total score of FMS > 14, *n* = 12; anterior distance difference of SEBT < 4 cm, *n* = 14; *P* > 0.05).

**Figure 1:**
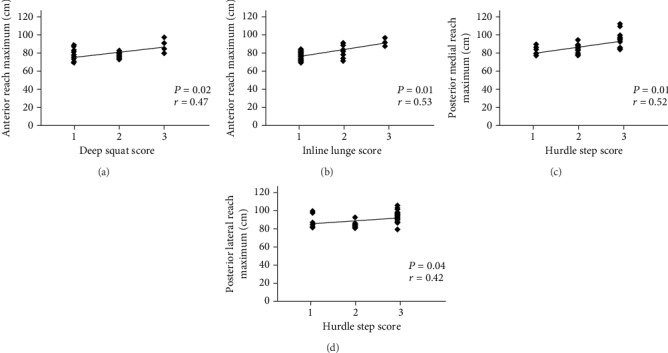
Significant correlations of anterior reach maximum in the SEBT with (a) deep squat and (b) inline lunge in the FMS. A significant correlation of hurdle step in the FMS with (c) posterior medial and (d) posterior lateral reach maximum in the SEBT.

**Figure 2:**
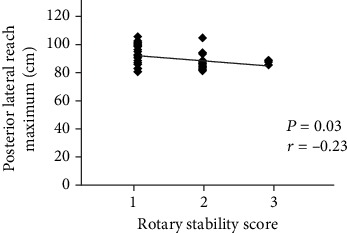
A significant correlation of posterior lateral reach maximum in the SEBT with rotary stability in the FMS.

Table 3 shows a significant positive correlation between the anterior reach maximum of the SEBT and the deep squat and inline lunge of the FMS; the correlation was fair (*r* = 0.47, *P* = 0.02) and moderate-to-good (*r* = 0.53, *P* = 0.01, Figure 1), respectively. Moreover, the posterior medial and posterior lateral reach maximums of the SEBT also indicated a significant positive correlation with the hurdle step of the FMS; this correlation was moderate-to-good (*r* = 0.52, *P* = 0.01) and fair (*r* = 0.42, *P* = 0.04, Figure 1), respectively. A significant negative correlation was revealed between the posterior lateral reach maximum of the SEBT and the rotary stability of the FMS (*r* = −0.23, *P* = 0.03, Figure 2).

The agility *T* test demonstrated a significant moderate-to-good negative correlation with the FMS trunk stability push-up; this correlation was *r* = −0.57 and *P* = 0.006 (Table 4). Moreover, the agility *T* test also indicated a significant fair negative correlation with the anterior reach maximum of the SEBT (*r* = −0.42, *P* = 0.04, Table 5). No significant correlations were observed between the vertical jump and FMS or SEBT components (*P* > 0.05).

## 4. Discussion

This study was designed to determine the relationship between FMS, SEBT, agility *T* test, and vertical jump test results and compared the differences between groups with high and low risks of sports injuries. The current results from this study found that partial items in the SEBT and FMS, particularly trunk stability and dynamic weight shifting abilities, exhibited significant correlations. Agility *T* test had significantly negative correlations with trunk stability push-up in FMS and anterior reach maximum in SEBT. However, there were no significant differences in FMS, SEBT, agility *T* test, and vertical jump between high and low risks of sports injuries.

Harshbarger et al. assessed the relationship between the FMS and SEBT scores in intercollegiate athletics [19]. SEBT results had a negligible relationship with FMS results. Nevertheless, correlations were discovered between rotary stability score in the FMS and anterior reach maximum (*r* = 0.41, *P* < 0.05) and posterior medial reach maximum scores in the SEBT (*r* = 0.31, *P* < 0.05) [19]. Harshbarger et al. suggested that both assessments required core muscle stability to maintain postural stability during the performance of extremity movements [19]. A study by Lockie et al. explored the relationship between the FMS and modified-SEBT results of collegiate athletes [11]. Scores for trunk stability push-up in the FMS had a fair correlation with those for posteromedial reach maximum (*r* = 0.37, *P* < 0.05) with the right stance leg and anteromedial reach maximum with the right (*r* = −0.33, *P* < 0.05) and left (*r* = −0.32, *P* < 0.05) stance legs of the SEBT. A fair correlation was revealed between scores for the inline lunge of the FMS and posteromedial reach maximum (*r* = 0.46, *P* < 0.05) with the right stance leg [11]. The outcomes expressed FMS and SEBT challenged the dynamic stability of athletes but did not further explore reasons of their relationship. The aforementioned results differed from the results of this study, as posteromedial and posterolateral excursions of the SEBT exhibited significant positive correlation with the hurdle step movement in our study findings. We suggested that the participant reduces the center of pressure to one leg for the contralateral leg crossover step during the hurdle step exercise. This movement pattern is similar to the posteromedial and posterolateral reach test in the SEBT. Another noteworthy finding in our study is that the rotary stability scores in FMS had a significantly negative relationship with that for posterolateral reach in the SEBT. In FMS, rotary stability refers to a trunk rotation movement, whereas posterolateral reach in the SEBT is an antirotation movement of core muscles. The negative relationship between these two movements was due to the use of reverse movement patterns.

The agility *T* test and vertical jump test are common athletic performance tests. Andersen et al. noted a significant correlation between muscle strength in the lower extremities of collegiate soccer players and agility (*r* = −0.67, *P* < 0.05) and vertical jump (*r* = 0.54, *P* < 0.05) [20]. Maggioni et al. indicated that jump performance was a potential factor to increase sprint ability [21]. Moreover, an increase of sprint ability could improve jump performance. Both abilities influenced each other and could be predictors of sport performance [22]. The agility *T* test and vertical jump scores of participants with a high risk of sports injury (FMS score ≤ 14; SEBT score difference < 4 cm) were not significantly different from those with low risk injury in this study. We conjectured that junior athletes had a sufficient level of sport fitness, suggesting that agility and muscle power were not factors related to injury. However, only agility *T* test scores exhibited significant correlations with scores for trunk stability push-up in the FMS (*r* = −0.57, *P* < 0.05) and anterior reach maximum in the SEBT (*r* = −0.42, *P* < 0.05), respectively. No relationships were observed between vertical jump, FMS, and SEBT scores. Better core muscle stability and dynamic balance control of anterior distance reflected to perform the task of agility *T* test. Armstrong and Greig postulated that in-line lunge in the FMS was a highlighted predictor of *T* test performance, resulting in a significant correlation with the performance of netball and rugby players [6]. They indicated that the relationships between the FMS, SEBT, and agility performance are sport-specific. This may also explain differences in the results of our study.

FMS scores less than or equal to 14 were associated with increased injury risk, although the sensitivity was low [23]. The risk of sports injury was 2.04 times higher among those with FMS scores less than or equal to 14. Even, injury risk was 4.2 times greater to combine with poor physical fitness [24]. Kiesel et al. obtained that FMS scores were obtained before the start of the season for 46 football players, and FMS ≤ 14 was found to positively predict serious injury (specificity = 0.91; sensitivity = 0.54). The odds of sustaining a serious injury were 11.7 times higher in those with FMS ≤ 14 compared with those with FMS > 14 [25]. Chorba et al. revealed that 69% of athletes scoring 14 or lower sustained an injury, with a sensitivity and specificity of 0.58 and 0.74 for all study participants. A significant correlation was identified between low-scoring athletes and injury (*r* = 0.76, *P* = 0.02) [26]. In the current study, 62.50% of junior athletes (*n* = 20) with an FMS score ≤ 14 had a higher risk of sports injury. The result of this study was higher than that reported by Smith et al., who revealed that 33% of high school male athletes had a high risk of sports injury (FMS score ≤ 14) [12]. Geographical and ethnic differences may be factors behind the differences in these different results. When junior athletes with high (FMS score ≤ 14) or low (FMS score > 14) risk of sports injury were compared, no significant differences were observed in SEBT, agility *T* test, and vertical jump. FMS is a functional movement test and used to identify deficits of exercise movement. FMS score is also predictive of sports injury and is able to assess musculoskeletal conditions. This study found that although participants with an FMS score of <14 had a high risk of sports injury, no differences were found among dynamic balance, agility, and vertical jump scores. The outcomes indicated that FMS cannot identify these intrinsic physical factors.

Gonell et al. demonstrated that a difference in the anterior distance between both lower extremities greater than 4 cm on the SEBT resulted in individuals being 2.5 times more likely to suffer sport injuries, such as an ankle sprain [27]. This finding related to poor dynamic balance could identify basketball players who are more susceptible to ankle injury. Moreover, high school girls with a lower normalized composite reach distance in the SEBT were 6.5 times more likely to sustain a sports injury in the lower extremities [15]. Grassi et al. indicated that the SEBT requires muscle strength of the lower extremities, coordination, and agility. This may increase the test's sensitivity and ability to predict sports injuries [28]. The SEBT could help basketball players to identify deficits in these areas and to improve their performance using a neuromuscular training program [29]. Assessing the neuromuscular characteristics and sports injury of the lower extremity in basketball players receiving neuromuscular training is useful [30, 31]. In the current study, 56.25% of junior athletes (*n* = 18) with an SEBT score difference of ≥4 cm had a high risk of sports injury. The findings indicated no significant differences among FMS, agility *T* test, and vertical jump test scores in groups with high (SEBT score difference ≥ 4 cm) and low (SEBT score difference < 4 cm) risks of sports injuries. The SEBT is a dynamic balance test and designed to identify physical performance. It is also used for predicting sports injury risks [32]. Similar to the FMS, however, these results indicated that the SEBT cannot identify functional movement, agility, or muscle power.

This study has some limitations. First, this study did not track the occurrence of sports injuries, and it was impossible to determine the correlation between sports injury events and these variables (i.e., FMS, SEBT, agility *T* test, and vertical jump test scores). Second, a comparison between variables across different age groups was not performed. We suggest that future studies target different races, geographic regions, and age groups to explore the relationship between FMS, SEBT, and physical fitness and to provide reference indicators for sports injury prevention.

## 5. Conclusion

Overall, junior athletes with an FMS score of ≤14 or an SEBT score difference of ≥4 cm have a higher risk of sports injury. However, the results did not reveal differences in FMS, SEBT, and physical fitness to compare with low and high risks of sports injury. Correlations between FMS and SEBT showed that the deep squat, hurdle step, inline lunge, and rotary stability exercises in the FMS had fair and moderate-to-good correlations with SEBT components, suggesting similar movement patterns were required to complete both tests. Anterior reach maximum of the SEBT and the trunk stability push-up of the FMS demonstrated fair and moderate-to-good correlations with the agility *T* test, respectively. These relationships may be due to similar task requirements of trunk stability and dynamic weight shifting abilities. We also suggest that exercises involving trunk stability and dynamic weight shifting ability could cover in training sessions. They could influence the functional performance, balance, and agility ability of junior athletes.

## Figures and Tables

**Table 1:** Outcomes of FMS, SEBT, agility *T* test, and power in all participants.

Items	Value (*n* = 32)
FMS	
Deep squat	1.68 ± 0.78
Hurdle step	2.32 ± 0.48
Inline lunge	1.45 ± 0.74
Shoulder mobility	1.64 ± 1.00
Active straight-leg raise	1.86 ± 0.35
Trunk stability push-up	2.32 ± 1.09
Rotary stability	1.50 ± 0.80
Total score	12.18 ± 2.02
SEBT	
Anterior reach maximum (cm)	78.15 ± 7.26
Posterior medial reach maximum (cm)	89.32 ± 8.39
Posterior lateral reach maximum (cm)	90.57 ± 6.94
Composite score (%)	85.98 ± 6.20
Agility *T* test (sec)	12.59 ± 1.33
Vertical jump (cm)	45.68 ± 7.77

**Table 2:** Scores for the FMS, SEBT, agility *T* test, and power by high and low risks of sports injury.

	FMS ≤ 14 (*n* = 20)	FMS > 14 (*n* = 12)	Difference ≥ 4 (*n* = 18)	Difference < 4 (*n* = 14)
FMS				
Deep squat	1.60 ± 0.75	2.50 ± 0.70	1.77 ± 0.92	1.56 ± 0.52
Hurdle step	1.70 ± 0.47	2.01 ± 0.51	1.69 ± 0.48	1.78 ± 0.44
Inline lunge	1.40 ± 0.75	2.02 ± 0.61	1.38 ± 0.76	1.56 ± 0.72
Shoulder mobility	1.60 ± 1.05	2.08 ± 0.42	1.85 ± 1.06	1.33 ± 0.86
Active straight-leg raise	1.85 ± 0.36	2.09 ± 0.55	1.85 ± 0.37	1.89 ± 0.33
Trunk stability push-up	2.25 ± 1.11	2.76 ± 0.31	2.31 ± 1.10	2.33 ± 1.11
Rotary stability	1.45 ± 0.82	2.08 ± 0.09	1.54 ± 0.87	1.44 ± 0.72
Total score	11.85 ± 1.78	15.50 ± 0.70	12.38 ± 2.21	11.89 ± 1.76
SEBT				
Anterior reach maximum (cm)	78.22 ± 7.49	77.44 ± 6.18	78.29 ± 5.93	77.93 ± 9.23
Posterior medial reach maximum (cm)	89.29 ± 8.80	89.54 ± 2.09	90.39 ± 5.05	87.76 ± 9.73
Posterior lateral reach maximum (cm)	90.73 ± 7.24	88.83 ± 2.93	91.43 ± 4.35	89.30 ± 9.22
Composite score (%)	86.02 ± 6.48	85.48 ± 2.39	86.61 ± 1.36	85.05 ± 8.40
Agility *T* test (sec)	12.65 ± 1.34	12.00 ± 1.41	12.77 ± 9.15	12.33 ± 1.32
Vertical jump (cm)	45.50 ± 8.01	47.50 ± 6.36	45.15 ± 7.53	46.44 ± 5.63

^∗^ *P* < 0.05; FMS: functional movement screen; SEBT: star excursion balance test.

**Table 3:** Correlations between items of FMS and SEBT.

FMS	SEBT		
	Anterior reach maximum	Posterior medial reach maximum	Posterior lateral reach maximum
Deep squat	0.47^∗^	0.14	-0.015
Hurdle step	0.16	0.52^∗^	0.42^∗^
Inline lunge	0.53^∗^	0.26	0.38
Shoulder mobility	-0.28	-0.19	-0.10
Active straight-leg raise	-0.28	-0.18	-0.16
Trunk stability push-up	0.25	0.19	0.06
Rotary stability	0.25	0.11	-0.23^∗^

^∗^ *P* < 0.05; SEBT: star excursion balance test.

**Table 4:** Correlations between items of FMS and scores of the agility *T* test and vertical jump.

FMS	Agility *T* test	Vertical jump
Deep squat	-0.17	0.12
Hurdle step	-0.14	0.06
Inline lunge	-0.10	0.06
Shoulder mobility	0.25	-0.33
Active straight-leg raise	-0.01	-0.03
Trunk stability push-up	-0.57^∗^	0.39
Rotary stability	-0.19	0.35

^∗^ *P* < 0.05; FMS: functional movement screen.

**Table 5:** Correlations between items of SEBT and scores of the agility *T* test and vertical jump.

SEBT	Agility *T* test	Vertical jump
Anterior reach maximum	-0.42^∗^	0.11
Posterior medial reach maximum	-0.14	-0.13
Posterior lateral reach maximum	0.02	-0.17
Composite score	-0.08	-0.14

^∗^ *P* < 0.05; SEBT: star excursion balance test.

## Data Availability

The data used to support the findings of this study are included within the article.
